# *Clostridium septicum*-infected Stanford type A acute aortic dissection: a case report

**DOI:** 10.1186/s40792-020-0770-y

**Published:** 2020-01-16

**Authors:** Kiyotoshi Akita, Yoshiyuki Takami, Kazuki Matsuhashi, Yusuke Sakurai, Kentaro Amano, Hiroshi Ishikawa, Tadahito Eda, Yasushi Takagi

**Affiliations:** Department of Cardiovascular Surgery, Fujita Health University School of Medicine, 1-98 Dengakugakubo Kutsukake-cho, Toyoake, 470-1192 Japan

**Keywords:** *Clostridium septicum*, Thoracic aortitis, Acute aortic dissection

## Abstract

**Background:**

Thoracic aortitis caused by *Clostridium septicum* is a rare infection with a strong association with malignancy and high mortality rate when left untreated. We report a case of surgical treatment for Stanford type A acute aortic dissection in a patient with *C. septicum* sepsis and thoracic aortitis.

**Case presentation:**

A 63-year-old hypertensive man with rheumatoid arthritis presented with general malaise and diagnosed with *C. septicum*-infected aortitis with sepsis. On the 5th day of hospitalization, Stanford type A acute aortic dissection developed with severe aortic regurgitation. The patient underwent emergent surgical treatment successfully with excision of the infected ascending aorta and aortic root followed by replacement using a composite graft, followed by diagnosis of sigmoid colon cancer 7 months after aortic surgery. He was scheduled to undergo elective colon surgery.

**Conclusions:**

*C. septicum* aortitis can progress quickly, causing aneurysm or dissection. Therefore, in a patient with *C. septicum* aortitis, prompt surgical in situ graft replacement should be performed to debride the infected vascular lesions. Further investigations for gastrointestinal and hematological malignancies as a source of *C. septicum* should be also conducted.

## Introduction

*Clostridium septicum* is an anaerobic, gram-positive bacillus to withstand a variety of environments. It accounts for 1.3% of all clostridial infections, but its impact lies in its association with occult gastrointestinal and hematological malignancies [1–3]. We present a rare case of *C. septicum*-infected aortitis causing Stanford type A acute aortic dissection with sepsis, treated successfully with excision of the infected ascending aorta and aortic root followed by replacement with a composite graft. Subsequent evaluation confirmed a colonic malignancy, prompting early intervention. The patient’s consent was obtained for publication.

## Case presentation

A 63-year-old hypertensive man with rheumatoid arthritis controlled by methotrexate presented to the emergency department of the local hospital with consciousness disturbance, following general malaise. White cell count (WBC) was 32,000/μL, and C-reactive protein (CRP) was 30.0 mg/dL. The enhanced-computed tomography (CT) scans showed gas loculations tracking within the wall of the ascending aorta with a maximal diameter of 31 mm. He was transferred to our institute with the diagnosis of acute septic aortitis. Physical examinations revealed mild confusion, a high-grade fever of 39.1 °C, heart rate of 112 beats/min, blood pressure of 116/70 mm Hg, respiratory rate of 20 breaths/min, and oxygen saturations of 96% on room air. Biochemistry analysis also revealed low nutrition (albumin of 2.1 g/dL, triglyceride of 74 mg/dL, and total cholesterol of 74 mg/dL) and electrolyte disturbances (serum sodium of 122 mEq/L and chloride of 85 mEq/L).

**Fig. 1:**
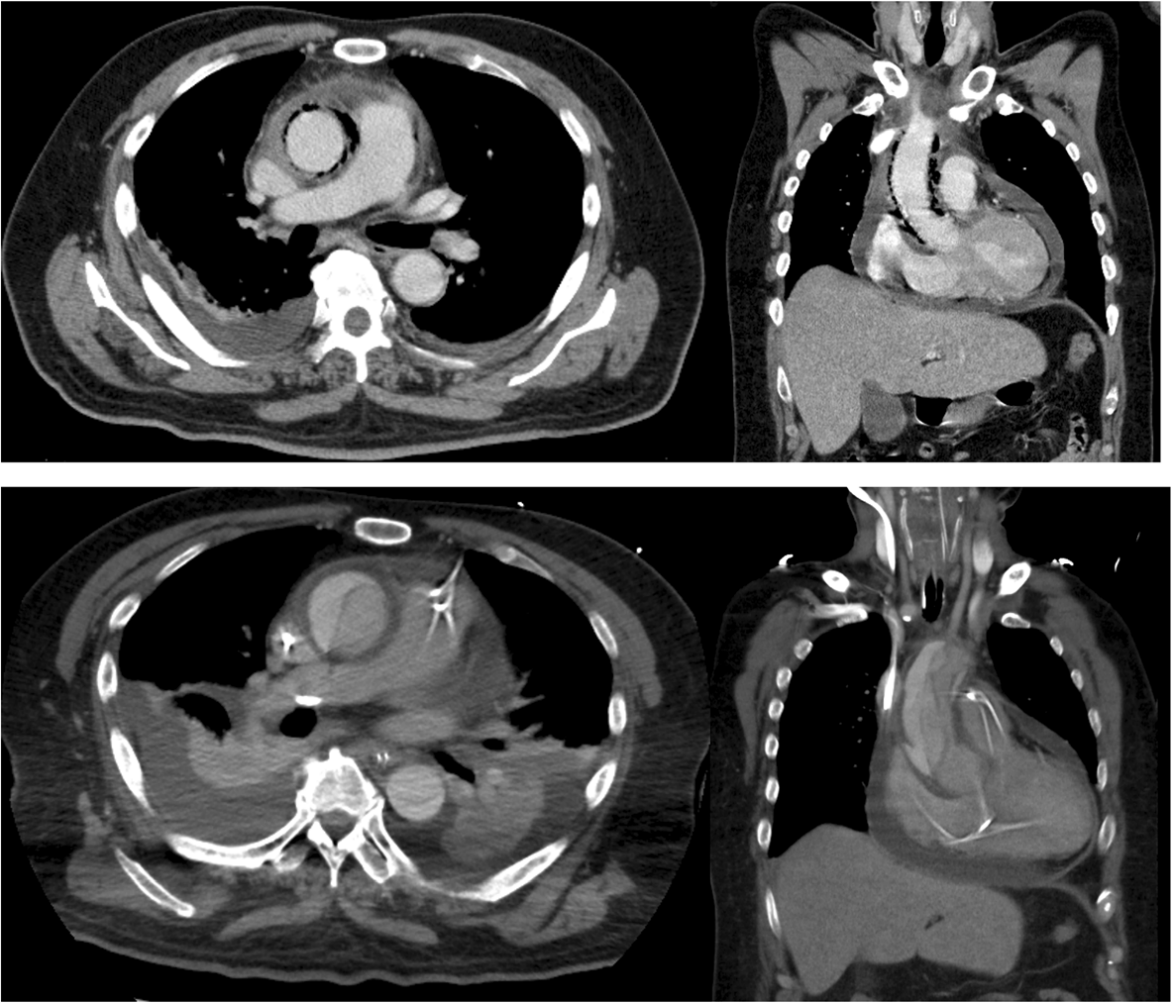
Enhanced-computed tomography (CT) images on the 3rd (upper) and 5th (lower) days of hospitalization

Emergency surgical intervention was not indicated because the patient was in too sick and poor conditions with high fever and poor nutrition for aortic surgery. In addition, the aorta was not dilated, and the small amount of pericardial effusion had no hemodynamic impact on the heart. We planned urgent surgery after improving the patient’s condition with intensive antibiotics and nutrition therapy, with careful follow-up CT scans. To treat severe systemic infection with unknown pathogens, tazobactam/piperacillin (TAZ/PIPC) of 4.5 g/day and vancomycin (VCM) of 1 g/day were started, according to the recommendation by the microbiologist. On the 3rd day of hospitalization, the CT showed neither changes of the gas image around the ascending aorta nor the diameters of aorta (Fig. 1), with improved data (WBC of 19400/μL, CRP of 29.4 mg/dL). Blood cultures were positive for *C. septicum* susceptible to TAZ/PIPC and VCM.

On the 5th day of hospitalization, the patient experienced sudden respiratory distress. Urgent echocardiography showed severe aortic regurgitation due to prolapse of the non-coronary cusp without any evidences of endocarditis. Urgent CT scans also revealed Stanford type A acute aortic dissection (Fig. 1).

Therefore, emergency surgery was performed via median sternotomy under general anesthesia. When the pericardium was incised, purulent discharge was found. The infection seemed to extend from the aortic root to the distal ascending aorta. Cardiopulmonary bypass (CPB) was started between the right axillary/femoral arteries and the right atrium. The left ventricle was vented through the right superior pulmonary vein. The patient was cooled down to 25 °C, followed by circulatory arrest with deep hypothermia. Antegrade selective cerebral perfusion was established by right axillary perfusion with a clamped brachiocephalic artery and direct cannulation of the left common carotid and subclavian arteries. Myocardial protection was achieved by antegrade and retrograde intermittent cold blood cardioplegia. An entry tear of the aortic dissection was observed just above the orifice of the left coronary artery. To exclude the entry near the orifice of the left coronary artery, to treat severe aortic regurgitation, and to eliminate the evident infected aortic tissue, we performed aortic root and ascending aortic replacement. The aortic arch did not appear to be severely infected and was not removed. The distal ascending aorta was transected circumferentially at the site close to the origin of the brachiocephalic artery. The dissecting lumen was reinforced with insertion of BioGlue® (CryoLife Inc., Kennesaw, NW, USA). The aortic stump was covered with a paired Teflon felt strip inside and outside the aorta, reinforced with continuous sutures, and anastomosed with a rifampicin-soaked 24 mm vascular graft (Gelweave®: Terumo, Tokyo, Japan). After distal anastomosis is completed, total body perfusion through the side branch of the graft was resumed by applying the clamp on the graft proximal to the anastomosis.

Since both coronary buttons were too fragile due to severe infection, we applied coronary artery bypass grafting for coronary reconstruction. After the infected tissue was resected, a composite graft consisting of a 21-mm mechanical valve (Regent®: Abbott, Chicago, IL) and a rifampicin-soaked Gelweave® was anastomosed to the aortic annulus with interrupted polypropylene pledgeted sutures. Then, two saphenous vein grafts were anastomosed to the right coronary and left anterior descending arteries, respectively, and the both coronary buttons were closed, followed by proximal anastomosis of the vein grafts.

The durations of operation, CPB, and cardiac arrest were 891, 521, and 338 min, respectively. Identification of the coronary arteries to be grafted was so difficult due to severe adhesions and thick peels around the heart that the times of CPB and cardiac arrest were long. As a result, hemodynamic instability with difficult weaning from CPB demanded circulatory assist with venous-arterial extracorporeal membrane oxygenation (ECMO) with the open sternum.

**Fig. 2:**
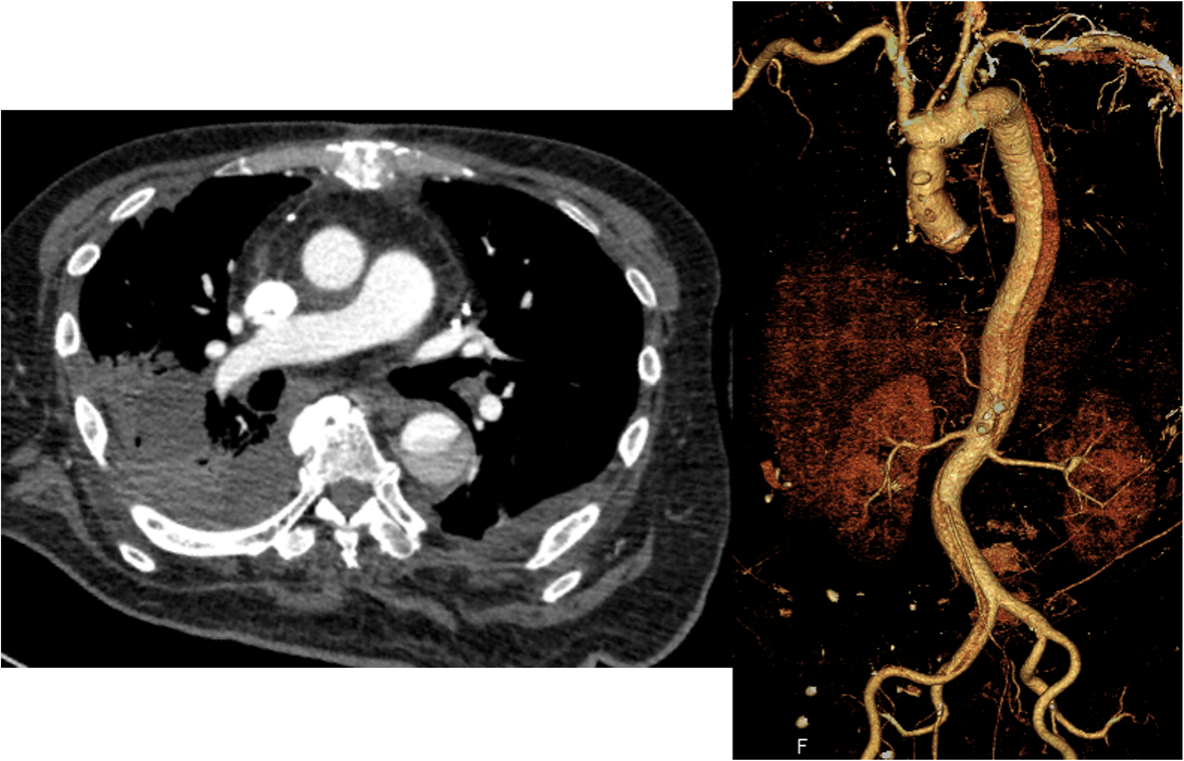
Postoperative enhanced-computed tomography (CT) images

*C. septicum* was also detected from the specimens of the infected arterial wall. After surgery, according to the recommendation by the microbiologist on the basis of sensitivity, TAZ/PIPC, and VCM were changed to meropenem. On the 4th postoperative day (POD), the patient was weaned from ECMO with stable hemodynamics while the sternum was still left open. We did not perform any lavages of pericardial cavity during the week with the open sternum. On the 8th POD, the sternum was closed with omental coverage of the area surrounding the prosthetic graft and aortic arch. On the 21st POD, the patient was extubated, followed by discharge from ICU on the 32nd POD. Postoperative CT scans showed no findings of infection around the prosthetic graft and residual aortic dissection of Debakey type IIIb with partial thrombus formation in the pseudolumen (Fig. 2). As inflammatory markers improved significantly, he was discharged from our hospital to the referral hospital for rehabilitation on the 77th POD with oral administration of levofloxacin and regular follow-up. At the referral hospital, the patient was still so sick that rehabilitation took time. Seven months after surgery when we finally obtain the informed consent for the endoscopy, he was diagnosed with sigmoid colon cancer and scheduled to undergo elective colon surgery.

## Discussion

*C. septicum* is a gram positive, spore-forming anaerobic gas-producing bacterium. Alpern et al. first reported a link between *C. septicum* infection and malignancy [1]. Koransky et al. also reported that 42 of 59 patients with *C. septicum* bacteremia had malignancy [2]. Thus, Katlic et al. stressed that in a patient with *C. septicum* bacteremia, malignancy should be sought and that anaerobic as well as aerobic cultures should be obtained in the septic patient with known malignancy [3].

We report a rare patient with *C. septicum sepsis* causing emphysematous aortitis, resulting in Stanford type A acute aortic dissection, who underwent surgical treatment successfully, followed by diagnosis of sigmoid colon cancer 7 months after aortic surgery.

There were several reports of *C. septicum*-infected aortitis, associated with high mortality up to 80% [4–6]. According to them, the sites of aortitis included ascending/arch (*n* = 15), descending (*n* = 6), thoracoabdominal (*n* = 6), and abdominal (*n* = 20). However, thoracic aortitis causing Stanford type A aortic dissection, as in our case, is extremely rare. Since our patient’s blood pressure was within normal ranges, the reason of acute dissection in this case was not hypertension but tissue fragility due to *C. septicum* aortitis, as in the literature review [6]. Among 15 patients with *C. septicum-*infected ascending aorta/arch aortitis, as in our case, 5 died with diagnosis of aortitis at autopsy, 3 underwent colon surgery but not aortic surgery (all died), 3 underwent vascular surgery but not cancer surgery (1 died and 2 survived), and 4 underwent aortic surgery followed by colon surgery, as in our case, all of whom survived. All survived patients underwent in-situ grafting, neither axillobifemoral bypass nor endovascular repair [4–6]. No patients underwent cancer surgery followed by aortic surgery.

We should learn the following lessons from our case and previous reports. First, when *C. septicum* is isolated in the blood culture, investigations for gastrointestinal cancer as a source of *C. septicum* should be conducted, although, in our case, malignancy should have been sought much earlier. At the same time, CT scans should be checked for the presence of periaortic gas, which is frequently concomitant with *C. septicum* aotitis. Second, aortic surgery, in situ graft replacement should precede cancer surgery in patients with *C. septicum*-infected thoracic aortitis. Third, since *C. septicum* aortitis can progress quickly, causing aneurysm or dissection, prompt surgical treatment should be performed. In the presented case, we should have operated a little earlier, before the aortic dissection occurred.

Finally, we should reflect on the use of levofloxacin at follow-up after surgery. A recent large epidemiological study found fluoroquinolone use, including levofloxacin, was associated with an increased risk of aortic aneurysm or dissection [7]. We should use levofloxatin more carefully for a while on the balance between the risk and benefit.

## Conclusion

*C. septicum* aortitis can progress quickly, causing aneurysm or dissection. Therefore, in a patient with *C. septicum* aortitis, prompt surgical in situ graft replacement should be performed to debride the infected vascular lesions. Further investigations for gastrointestinal and hematological malignancies as a source of *C. septicum* should be also conducted.

## Data Availability

Not applicable.

## Abbreviations

CPB: Cardiopulmonary bypass
CRP: C-reactive protein
CT: Computed tomography
ECMO: Extracorporeal membrane oxygenation
POD: Postoperative day
TAZ/PIPC: Tazobactam/piperacillin
VCM: Vancomycin
WBC: White cell count
